# Factors Influencing Epigenetic Mechanisms: Is There A Role for Bariatric Surgery?

**DOI:** 10.3390/ht9010006

**Published:** 2020-03-20

**Authors:** Alessio Metere, Claire E. Graves

**Affiliations:** 1 Surgical Sciences Department, “Sapienza” University of Rome, Viale Regina Elena 261, 00161 Rome, Italy; 2 Department of Surgery, University of California, San Francisco, 1600 Divisadero St., San Francisco, CA 94115, USA; claire.graves@ucsf.edu

**Keywords:** epigenetics, bariatric surgery, obesity, gene regulation

## Abstract

Epigenetics is the interaction between the genome and environmental stimuli capable of influencing gene expression during development and aging. A large number of studies have shown that metabolic diseases are highly associated with epigenetic alterations, suggesting that epigenetic factors may play a central role in obesity. To investigate these relationships, we focus our attention on the most common epigenetic modifications that occur in obesity, including DNA methylation and post-translational modifications of histones. We also consider bariatric surgery as an epigenetic factor, evaluating how the anatomic and physiologic modifications induced by these surgical techniques can change gene expression. Here we discuss the importance of epigenetic mechanisms in chronic disease and cancer, and the role of epigenetic disturbances in obesity, with a focus on the role of bariatric surgery.

## 1. Introduction

The concept of epigenetics was first outlined by Conrad Waddington in the early 1940s [1], who defined epigenetics as “the branch of biology that studies the causal interactions between genes and their products that give rise to the phenotype”. Epigenetic mechanisms change the regulation and expression of genes through, for example, methylation and histone modifications, but do not affect the DNA sequence [2]. Traditionally, the individual genes that constitute a particular genotype have been considered of fundamental importance, but the same dignity should be reserved for epigenetics. The genes inside a cell represent only the *potential* characteristics that the cell, and therefore, the organism may express, but the actual morphologic and functional features, the “phenotype”of the cell and organism, is determined by the expression of those genes. Consider the differences existing between some cells, for example a myocyte and a neuron, which are completely heterogeneous in terms of morphology, structure, and function, despite having the same genotype. It is evident, therefore, that gene expression and all the mechanisms governing gene expression are fundamental for the proper differentiation and function of a cell. 

Alterations at the level of gene expression, called by some authors epigenetic “mutations”, can be considered very similar to genetic modification, since they transform cellular behavior [3]. These epigenetic mechanisms play a crucial role in certain pathologies like cancer, where the untimely expression or activation of certain genes is responsible for the alteration of differentiation and replication mechanisms. Therefore, in light of the growing research that correlates epigenetics to multiple diseases, we should no longer consider certain pathologies as the exclusive consequence of DNA mutations. In fact, some diseases are also the result of epigenetic mutations, induced by environmental [4] or chemical agents [5,6], which ultimately alter cellular function. 

Because the preponderance of research in the field of epigenetics has focused on its role in carcinogenesis, this review will begin by describing the epigenetic mechanisms known to be involved in cancer, prior to discussing epigenetics in obesity and the unique role that bariatric surgery may play as an epigenetic factor. 

## 2. Epigenetic and Genetic Mechanisms for the Regulation of Gene Expression

Like DNA mutations, epigenetic mechanisms play a significant role in carcinogenesis. Epigenetic mechanisms utilize a variety of different strategies to regulate gene expression, generally through DNA methylation or histone modification [7]. Gene silencing can occur through the action of non-coding RNAs (miRNAs) or DNA methylation, the addition of a methyl group (via DNA methyltransferases) to cytosine residues in CpG islands in the promoter region of a gene [8]. These mechanisms alter physical accessibility to the regions of the genome where proteins and enzymes associated with gene expression bind, thereby preventing gene translation. Histone modifications are another means of epigenetic regulation. Histone acetylation via histone acetyltransferases, and deacetylation via histone deacetylases, are modifications that increase or decrease the transcription of genes, respectively. 

The environmental factors that can promote epigenetic mutations in humans are similar to known risk factors for cancer, including diet, lifestyle, and exposure to toxic substances. Many substances present in food, such as polyphenols or selenium (antioxidants present in fruits and vegetables), act precisely on DNA methylation [9,10]. Others, such as butyric acid, found in large quantities in cheeses, or sulforaphane, found in broccoli [11], can modify histones by inhibiting histone deacetylase [12]. Curcumin is a spice used in cosmetics, food flavoring, and food coloring, which is known to exert antioxidant effects. However, at high doses, it may produce toxic and carcinogenic effects in healthy cells. The cytotoxic effects observed after curcumin treatment may be due to post-translational modifications of histones, which lead to modifications in gene expression [13]. Additionally, a diet rich in fat can be associated with hypermethylation of specific DNA promoters of tumor suppressor genes [14]. All of these substances can influence epigenetic mechanisms, indicating that these molecules have an effect on the promotion or prevention of cancer. Other molecules, generally used for therapeutic purposes, are also able to alter gene expression through epigenetic mechanisms. The most striking example is diethylstilbestrol, a drug used in the past by pregnant women to prevent spontaneous abortions. Diethylstilbestrol has been shown to induce an increased risk of breast cancer and vaginal adenocarcinoma, as well as some reproductive abnormalities. These toxic effects are likely the consequence of epigenetic mechanisms, as diethylstilbestrol has been shown to alter the expression of DNA methyltransferase and genomic DNA methylation [15]. 

## 3. Epigenetic Factors and Environment

Not only can diet and drugs can alter epigenetic mechanisms, but the external environment can also play a role. Environmental pollutants, such as chromium, cadmium, nickel, mercury, and arsenic, are all thought to alter the epigenetic machinery. In particular, it has been shown that mercury exposure, for example, through dietary intake or environmental contact, may generate epigenetic alterations that lead to multiple deleterious health effects, such as atherosclerosis, myocardial infarction, adverse behaviors, and decreased newborn cerebellar size [16]. Analysis of renal tissue in mercury-treated animals showed significant damage due to the overactivity of proteolysis matrix metalloproteinase 9 (MMP9) caused by demethylation of the MMP9 regulatory region [17]. Ultraviolet radiation has been shown to reduce methylation levels through the inhibition of DNA methyltransferase activity [18]. Smoking has also been shown to affect methylation of tumor suppressor genes, both in human case–control studies [19] and in experiments conducted on mice [20]. Aberrant methylation of gastric mucosal genes is a common finding in humans infected with *Helicobacter pylori* and is an early event in gastric carcinogenesis [21]. The change in the epigenetic profile in response to infection may play a role in the development of related immune disorders and tumors previously associated with infectious agents [22]. Importantly, some epigenetic modifications acquired during pregnancy can be transmitted from generation to generation, though fertilization usually removes DNA methylation.

## 4. Epigenetics and Bariatric Surgery

As previously described, changes in the epigenetic profile can occur due to the external environment or exposure to factors like diet, drugs, food, or cigarettes, that are capable of altering the epigenetic state. Recently, it has been proposed that epigenetic mechanisms may also be related to the development of obesity [23]. Several studies have demonstrated that exercise and nutritional status induce acute changes in DNA methylation patterns in human skeletal muscle [24] and adipose tissue [25], therefore remodeling the epigenome of somatic tissues. In a recent study by Sergi Sayols-Baixeras et al. [26] investigating the association between DNA methylation and obesity traits using an epigenome-wide approach, 95 CpGs were associated with obesity traits like body mass index (BMI) and waist circumference. Wahl et al. [27] also used epigenome-wide association to link BMI to changes in DNA methylation and suggested through genetic association analyses that these changes are the consequence of adiposity rather than the cause. Moreover, they found that changes in DNA methylation predict future development of type 2 diabetes. Additionally, Keller et al. [28] showed that in vitro promoter methylation of specific obesity-related genes represses the transcriptional activity of the gene-reporter constructs, providing functional evidence that methylation in these genes directly affects gene activity.

Based on these findings, we could also consider bariatric surgery as an “environmental factor” related to obesity and nutritional status, capable of changing the epigenetic profile. In particular, bariatric surgery induces anatomic, and consequently, physiologic modifications that could change gene expression [29]. Bariatric surgery consists of several surgical procedures for the treatment of obesity, indicated for patients with a BMI higher than 40 kg/m^2^, or higher than 30 kg/m^2^ in the presence of other comorbidities. These procedures cause weight loss through restriction, i.e., limiting the volume of food the stomach can hold, or malabsorption, by re-routing food to decrease intestinal surface area, or through a combination of these mechanisms. The most commonly performed bariatric surgery procedures are the Roux-en-Y gastric bypass (RYGB), sleeve gastrectomy, and biliopancreatic diversion with duodenal switch. Each procedure has different technical advantages and disadvantages, as well as different risks and potential complications, but all are proven to induce weight loss. 

Romain Barres et al. [30] were among the first authors to study the relationship between epigenetic factors and bariatric surgery, showing remodeling of promoter methylation induced by weight loss after RYGB. Specifically, they demonstrated that promoter methylation of PGC-1α and PDK4, two genes involved in mitochondrial function and fuel utilization in skeletal muscle, is altered with obesity and subsequently restored to non-obese levels after RYGB-induced weight loss. Surgery-induced weight loss has also been associated with remodeling of sperm DNA methylation, notably at genetic locations implicated in the central control of appetite [31]. However, Coppedè et al. [32] demonstrated that DNA methylation of genes encoding for molecules regulating appetite, food intake, or obesity does not successfully predict weight loss following Roux-en-Y gastric bypass. 

Epigenetic mechanisms also appear to be involved in the progression of non-alcoholic fatty liver disease (NAFLD), a liver disorder ranging from simple hepatic steatosis to inflammatory non-alcoholic steatohepatitis, fibrosis, and cirrhosis, commonly associated with obesity. Several differentially methylated regions have been associated with metabolic pathways, depending on the degree of liver fibrosis, which are reversed after bariatric surgery-induced weight loss [33]. Additional research [34] found significant changes in the DNA methylation of subcutaneous adipose tissue and omentum after gastric bypass and weight loss, including differential modification of obesity genes. In an epigenome-wide association analysis, Fraszczyk et al. [35] revealed 4857 differentially methylated CpG sites 12 months after bariatric surgery. All of this data provides growing evidence that obesity and bariatric surgery-induced weight loss have a dynamic effect on the epigenome.

## 5. Discussion

Interest in epigenetics, and its role in the development of cancer and chronic diseases, is rapidly growing. The contribution of epigenetics to obesity remains unknown. A meta-analysis of genome-wise association studies (GWAS) for BMI by Yengo et al. [36], with a total sample size of approximately 700,000 participants, found 941 BMI-associated single nucleotide polymorphisms (SNPs), accounting for approximately 6% of variance in BMI. This meta-analysis more than doubled the sample size of previous GWAS of BMI [37], and discovered hundreds of new loci, suggesting that as sample sizes continue to grow, so will our insight into the role of genetics in obesity. However, as previously discussed, the influence of environmental factors such as diet and exercise on obesity is undeniable, and it is these non-genetic factors that induce alterations in an individual’s epigenetic profile [24,25].

The study of bariatric surgery may be uniquely beneficial to tease apart the genetic vs. epigenetic contributions to obesity and to elucidate the mechanisms of epigenetic factors involved in weight loss. Bariatric surgery permits us to directly measure the changes in DNA methylation or histone modification that occur with the significant weight loss induced by these techniques. The study of epigenetics in obesity and weight loss represents a huge potential opportunity to better clarify the epigenomic changes that occur in human disorders. The NIH has recently launched its Roadmap Epigenomics Program, with the aim of developing methods to analyze the epigenome and discover novel epigenetic markers of diverse human diseases [38]. The findings derived from these epigenetic studies could be revolutionary and may add new weapons to cure many diseases, including new drugs able to act on epigenetic mechanisms. Epigenetic-based therapy, or “epidrugs”, are terms coined by Berdasco and Esteller in their recent review that identified one potential area of focus for translational epigenetics research [39]. However, it is also essential to elucidate other potential mechanisms to modify gene expression, including clarifying the role of bariatric surgery. Further studies investigating the relationship between epigenetics, obesity, and bariatric surgery are required to expand the exciting results that are starting to emerge. Research in the field of epigenetics is only at the beginning of a long but undoubtedly fruitful road.
